# Bibliometric analysis of tuberculosis molecular epidemiology based on CiteSpace

**DOI:** 10.3389/fpubh.2022.1040176

**Published:** 2022-11-22

**Authors:** Mei-qin Zheng, Xi-xi Li, Rui Xu, Shuo Liu, Zhi-yong Rui, Zhen-yong Guo, Di Chen

**Affiliations:** ^1^Department of Pharmacy, Beijing Chest Hospital, Capital Medical University, Beijing Tuberculosis and Thoracic Tumor Research Institute, Beijing, China; ^2^Department of Pharmacy, Beijing Hospital, National Center of Gerontology, Institute of Geriatric Medicine, Chinese Academy of Medical Sciences, Beijing, China

**Keywords:** tuberculosis, molecular epidemiology, bibliometrics, CiteSpace, Web of Science

## Abstract

**Background:**

Tuberculosis is a communicable disease that is a major cause of ill health. Bibliometrics is an important statistical methodology used to analyze articles and other publications in the literature study. In this study, publications on molecular epidemiology were analyzed using bibliometric analysis. The statistical analysis of influential publications, journals, countries and authors was first conducted.

**Methods:**

The Web of Science database was searched for publications on the molecular epidemiology of tuberculosis with the keywords “tuberculosis” and “molecular epidemiology” in the title. The number of publications, citation analysis, co-authorship of the author, institution and country, keyword co-occurrence, and reference co-citations were analyzed.

**Results:**

A total of 225 journal articles were retrieved. The mean citation was 37.72 per article and 292.69 per year. The annual publications on molecular epidemiology fluctuated within a certain range in the past. Journal of Clinical Microbiology is the most published journal with 33 articles. RASTOGI N is the most prolific author with 11 articles. The top 1 research institution is Inst Pasteur Guadeloupe. Stratified by the number of publications, the USA was the most prolific country. It also cooperates closely with other countries. Burstness analysis of references and keywords showed that the developing research trends in this field mainly focused on “genetic diversity” and “lineage” during the past decade.

**Conclusion:**

The annual publications on tuberculosis molecular epidemiology fluctuated within a specific range in the past decade. The USA continues to dominate research output and funding. The exchange of expertise, ideas, and technology is of paramount importance in this field. More frequent and deeper cooperation among countries or institutions will be essential in the future.

## Introduction

Tuberculosis (TB) remains a serious global public health threat asserted by the World Health Organization (WHO) in 2021. There were an estimated 9.9 million new cases of TB worldwide in 2020. The number of TB cases in China was 842,000, ranks second in the world flowing India (1). Molecular epidemiology of tuberculosis is based on genotyping technology and combines traditional epidemiology with emerging biotechnology to elucidate the occurrence, development, epidemic, drug resistance, and transmission rules of tuberculosis at the molecular level (2).

To have a more comprehensive understanding of the prevalence and transmission of tuberculosis and to formulate effective control measures, new theories and methods are needed to apply to tuberculosis research. Bibliometrics is a discipline that uses mathematics, statistics and other measurements to examine the quantitative relationship and development law of all knowledge carriers, such as documents and document information systems, and to explore the dynamic characteristics of science. As a relatively new literature research method, bibliometric analysis can evaluate the development trend of research activities by qualitative and quantitative methods according to the information provided by the literature database, grasp the development of a particular field and provide information for comparing the contributions of different levels (3). The bibliometric method can be used to analyse number of publications to efficiently find influential publications, authors, journals, organizations and countries. At present, there are a lot of software tools proposed to develop science mapping analysis (4–6). CiteSpace is a bibliometric tool based on the principle of “co-citation analysis theory” to reveal new technologies, hotspots, and trends in the medical field (7). It can perform bibliometric analysis on a specific area and draw a series of visual knowledge maps to explore the key paths and frontier developments in the evolution of scientific research fields.

In this paper, a bibliometric method is used to conduct a statistical analysis of English literature related to molecular epidemiology, and CiteSpace software is used to visualize the statistical results, which can help scholars quickly understand the research status and hotspots and provides new ideas and directions for studying the epidemic and transmission laws of tuberculosis.

## Data and methods

### Data collection

We used the keywords “tuberculosis” and “molecular epidemiology” in the title to retrieve manuscripts published from the Web of Science Core Collection from the establishment of the database to 2022. We collected 225 manuscripts and downloaded all information on 15 August 2022. The inclusion criteria were: (1) peer-reviewed published original articles; (2) reviews; (3) language in English, excluding meetings, letters, editorial material, books, and abstract.

### Data analysis and visualization

Annual publications, citations, funding agencies and source journals were imported into Microsoft Excel 2016 for analysis and visualization. Other information from 225 manuscripts was converted to a text file and then imported into CiteSpace 6.1R3 Basic. We analyzed co-authorship of the author, institution and country, keyword co-occurrence, and reference co-citations. The main parameters were set as follows: the time slicing was 1 year per slice from January 1993 to August 2022. The selection criteria were g-index and the scale factor was *k* = 25.

## Results

### Annual publications and citations

**Figure 1:**
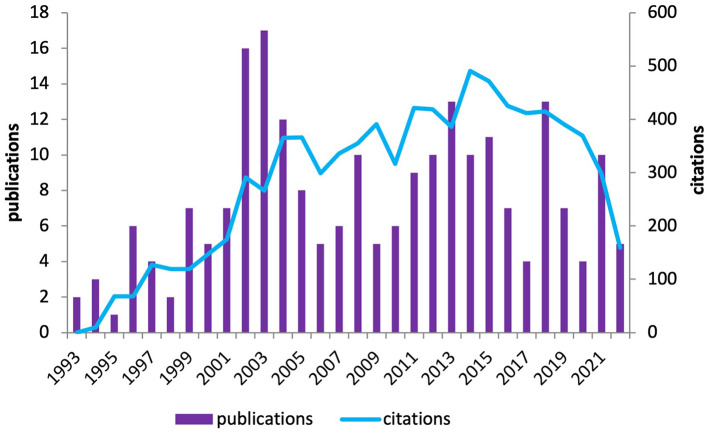
Times cited and publications on TB molecular epidemiology over time.

There were 197 articles and 28 reviews published between 1993 and 2022. The summed citations were 8,488. The average number of citations per item was 37.72 and the average number of citations per year was 292.69 (Figure 1).

The first article focus on the molecular epidemiology of tuberculosis was published in 1993. From 1993 to 2003, the number of publications increased year by year. The number of citations also increased fast, showing a rapid development trend. From 2004 to 2013, the research superheat in this field declined, but still showed an overall volatile increased trend. There were two peaks in 2008 and 2013, and the number of citations in this field reached a peak of 491 in 2014. The annual publications declined since 2014. In 2018 and 2021, there were two publication peaks, with 13 and 10 articles published, respectively, and the number of citations was declining year by year.

### Journal analysis

Articles in this field are mainly published in 92 journals, of which the productivity and average impact factors in the past 5 years of the top 10 active journals are shown in Table 1. Journal of Clinical Microbiology is the most published journal in this field with 33 articles. The average impact factor of the journal in the past 5 years is 8.075, and an impact factor in 2021 is 11.677. The second-ranked journal is the International Journal of Tuberculosis and Lung Disease, with a total of 22 published articles, with an average impact factor of 2.669 in the past 5 years and an impact factor of 3.427 in 2021.

**Table 1:** Productivity and average impact factors (in the past five years) of top 10 active journals.

**Journals**	**Numbers**	**Impact** **factor**
Journal of clinical microbiology	33	8.075
International journal of tuberculosis and lung disease	22	2.669
Plos ONE	16	4.069
Infection genetics and evolution	12	3.656
BMC infectious diseases	10	3.714
Clinical microbiology and infection	8	10.298
Emerging infectious diseases	7	10.717
Tuberculosis	5	2.973
Clinical infectious diseases	4	15.446
Journal of infection	4	19.923

### Funding agencies analysis

Table 2 shows top 10 of the most distributed funding agencies of tuberculosis molecular epidemiology. America plays an important role in supporting to research on tuberculosis molecular epidemiology with 24 studies and 23 studies supported by United States Department Of Health Human Services and National Institutes Of Health besides two funding agencies in the United States. Agencies in Europe take second place including three agencies in Britain and 1 agency of European Union, which support more than 28 projects. In this field, Canadian Institutes of Health Research in Canada and Conselho Nacional De Desenvolvimento Cientifico E Tecnologico in Brazil subsidize four researches, respectively.

**Table 2:** Top 10 funding agencies of tuberculosis molecular epidemiology.

**Funding agencies**	**Numbers**
United States department of health human services	24
National institutes of health Nih Usa	23
European commission	15
Nih national institute of allergy infectious diseases Niaid	15
Nih fogarty international center Fic	6
Wellcome trust	5
Canadian institutes of health research Cihr	4
Conselho nacional de desenvolvimento cientifico e tecnologico Cnpq	4
Medical research council Uk Mrc	4
Uk research innovation Ukri	4

### Co-author, co-institution, and co-country analysis

**Figure 2:**
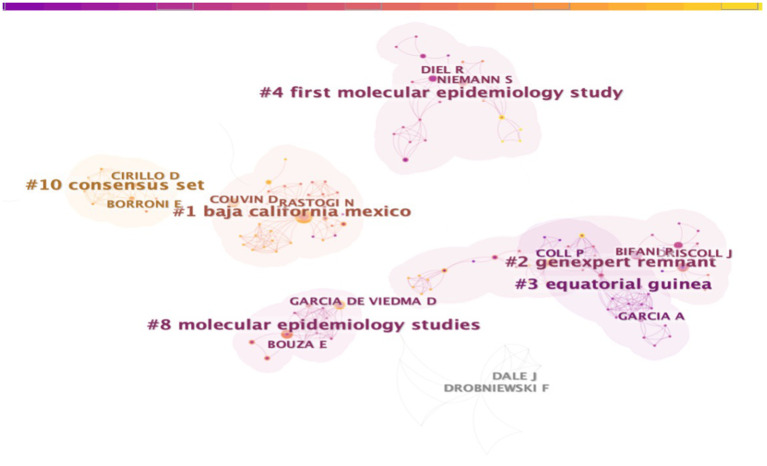
Clustered network of co-author analysis on tuberculosis molecular epidemiology.

**Figure 3:**
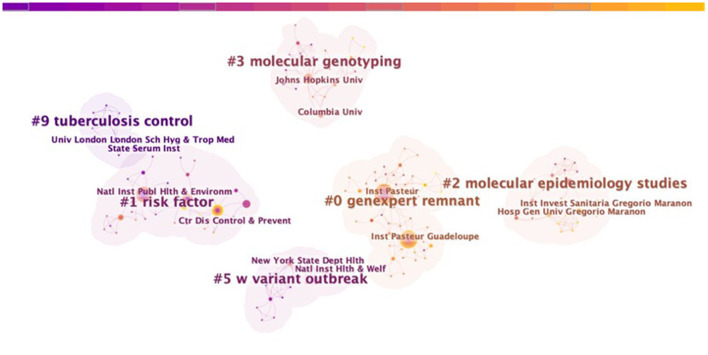
Clustered network of co-institution analysis on tuberculosis molecular epidemiology.

**Figure 4:**
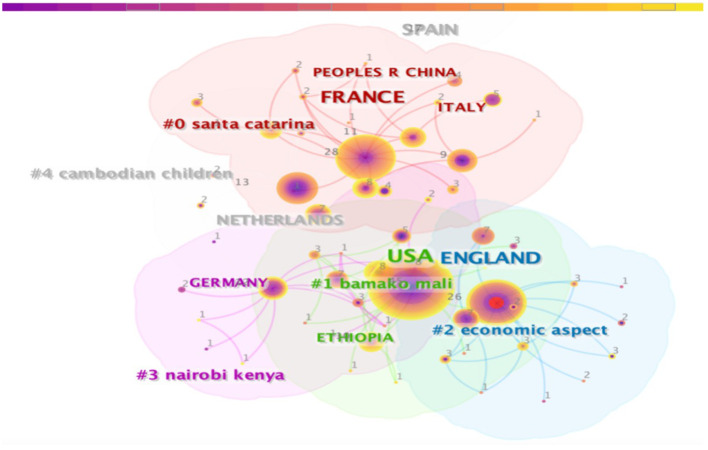
Clustered network of co-country analysis on tuberculosis molecular epidemiology.

Figures 2–4 show the cooperation network clustering map of the author, institution, and nation in the field of tuberculosis molecular epidemiology, respectively. The nodes in the figures represent the authors, institutions, or countries. The larger the node, the more published articles are. The lines between the nodes demonstrate the cooperative relationship between them. The colors of the nodes, lines, and cluster outlines in Figures 2, 3 are categorized by year. Purple indicates a distant age and yellow indicates a relatively recent age. The color of the node in Figure 4 shows the year, and the color of the connection line and cluster outline are used to distinguish different clusters. The nodes of the same cluster denote that their research fields are similar, and # are the cluster names extracted from the analyzed literature. Cluster names were extracted from the titles of the included literature using the log-likelihood ratio (LLR) method.

The top 5 prolific authors in the field of TB molecular epidemiology are RASTOGI N (11), DROBNIEWSKI F (10), ASEFFA A (5), AMENI G (5), and NIEMANN S (5). The numbers in parentheses are the corresponding publications. Figure 2 shows the top two authors who have published more papers in each cluster. The node of RASTOGI N is the largest and cooperates closely with other authors. It forms a complex academic cooperation network with COUVIN D (4) and others by working on the molecular epidemiology of tuberculosis in Baja California Mexico. In recent years, BORRONI E (2) and CIRILLOD (2) have collaborated on a consensus set.

The top 5 institutions in the field of tuberculosis molecular epidemiology and their publications shown in parentheses are Inst Pasteur Guadeloupe (12), Inst Pasteur (11), Natl Inst Publ Hlth & Environm (9), Ctr Dis Control & Prevent (7), Minist Hlth (6). Figure 3 shows the top two institutions with more publications in each cluster. Represented by Inst Pasteur Guadeloupe and Inst Pasteur, the largest academic cooperation system in this field has been formed in recent years to jointly carry out research related to gene expert remnant in the early years, Natl Inst Publ Hlth & Environm, Ctr Dis Control & Prevent, and Minist Hlth formed the second largest cooperative system to jointly study risk factors.

Figure 4 shows that USA has published a maximum of 45 papers and cooperated with ETHIOPIA (10). FRANCE ranks second in the number of published papers with 28 papers. At the same time, it has formed the largest cooperative group in this field with PEOPLESRCHINA (11), ITALY (9), and other countries. ENGLAND is the third country with 26 articles published and cooperated with RUSSIA (7), NORWAY (7), and other countries.

### Co-occurring keywords analysis

**Figure 5:**
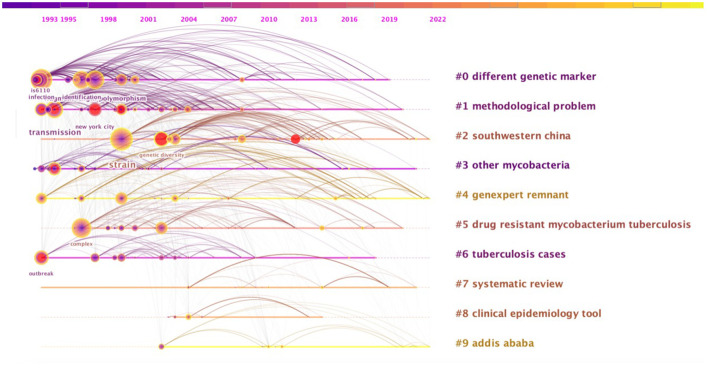
Clustered analysis of co-occurring keywords on tuberculosis molecular epidemiology.

By extracting co-occurring keywords from the selected literature, it can reflect the development trend and research hotspots of a certain field. In this study, 391 co-occurring keywords were extracted from the keywords of the 225 included literature. Ten clusters marked with # were extracted from the title of the included literature by LLR method cluster analysis, and the connecting line in the figure indicates that two keywords appear together in the same article. Figure 5 shows the top 10 high-frequency co-occurrence keywords except for search terms and the co-occurrence frequency is shown in parentheses, which are strain (78), transmission (62), fragment length polymorphism (37), new york city (34), identification (31) infection (31), complex (29), genetic diversity (28), is 6,110 (27), breakout (25). The color of the nodes in the figure is distributed by year, purple is the oldest, and yellow is the newest; red is the outbreak keyword in this field, which means it is a research hotspot in this field. It can be observed from Table 3 that the frequency of genetic diversity suddenly increased between 2007 and 2017, and it is the co-occurrence keyword with the strongest outbreak intensity in the field of TB molecular epidemiology research. Lineage has gradually increased in frequency since 2012, with an outbreak intensity of 4.67, ranking fourth, and the outbreak continues to this day. Figure 5 also shows the migration and changes of the 10 cluster names and the content of research hotspots over time.

**Table 3:** Top 10 keywords with the strongest citation bursts of tuberculosis molecular epidemiology.

**Keywords**	**Strength**	**Begin**	**End**	**1993–2022**
Human immunodeficiency virus	4.96	1993	2001	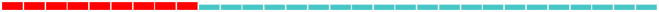
Tool	4.42	1993	2001	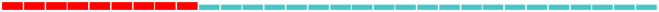
Element	4.31	1993	2001	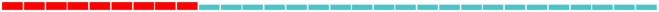
Outbreak	3.91	1993	2002	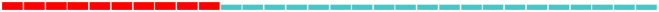
New york city	6.22	1998	2006	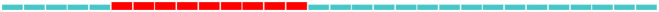
Transmission	3.69	1998	2004	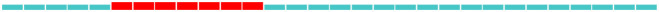
Sanfrancisco	4.30	2002	2006	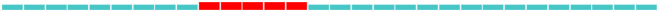
Pulmonary tuberculosis	3.58	2004	2006	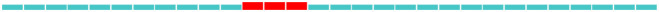
Genetic diversity	6.27	2007	2017	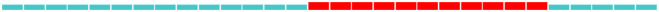
Lineage	4.67	2012	2022	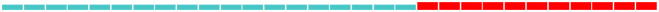

### Co-citation references analysis

**Figure 6:**
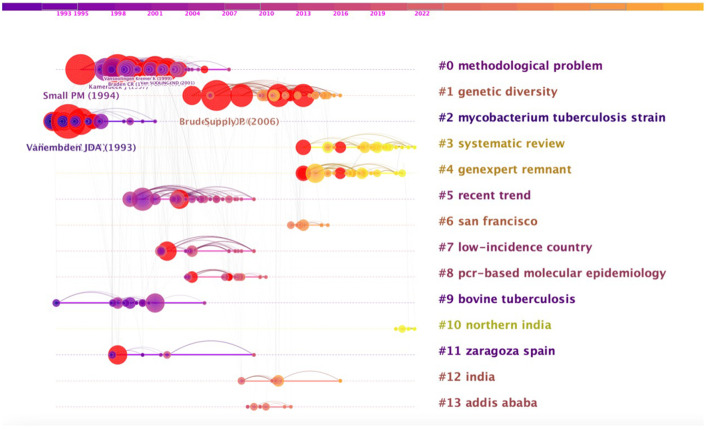
Clustered analysis of co-citation references on tuberculosis molecular epidemiology.

Co-citation analysis of references is the most distinctive feature of CiteSpace. The more documents in the cluster, the more important it represents the clustering field. Through the span of each document, we can see the rise, prosperity, and decline of a particular clustering research field. A total of 4,964 references were included in the 225 articles. Figure 6 extracts 825 of the co-cited documents as nodes to draw a sequence diagram. The 14 cluster names marked with # shown in the figure are the basic knowledge of TB molecular epidemiology and its deduction process over time. The top 10 references by co-citation frequency, author, publication journal and year, and co-citation frequency information are shown in Table 4.

**Table 4:** Top 10 co-cited references of tuberculosis molecular epidemiology.

**Title**	**Author**	**Journal**	**Year**	**Citations**
Transmission of tuberculosis in New York City. An analysis by DNA fingerprinting and conventional epidemiologic methods	D Alland	N Engl J Med.	1994	36
The epidemiology of tuberculosis in San Francisco. A population-based study using conventional and molecular methods	Small PM	N Engl J Med.	1994	35
Simultaneous detection and strain differentiation of Mycobacterium tuberculosis for diagnosis and epidemiology	J Kamerbeek	J Clin Microbiol.	1997	30
Mycobacterium tuberculosis complex genetic diversity: mining the fourth international spoligotyping database (SpolDB4) for classification, population genetics and epidemiology	Brudey K	BMC Microbiol.	2006	28
Strain identification of Mycobacterium tuberculosis by DNA fingerprinting: recommendations for a standardized methodology	van Embden JD	J Clin Microbiol.	1993	24
Proposal for standardization of optimized mycobacterial interspersed repetitive unit-variable-number tandem repeat typing of Mycobacterium tuberculosis.	Supply P	J Clin Microbiol.	2006	22
Molecular epidemiology of tuberculosis in the Netherlands: a nationwide study from 1993 through 1997	van Soolingen D	J Infect Dis.	1999	21
Interpretation of restriction fragment length polymorphism analysis of Mycobacterium tuberculosis isolates from a state with a large rural population.	Braden CR	J Infect Dis.	1997	19
Molecular epidemiology of tuberculosis and other mycobacterial infections: main methodologies and achievements.	Van Soolingen D	J Intern Med.	2001	18
Predominance of a single genotype of Mycobacterium tuberculosis in countries of east Asia.	van Soolingen D,	J Clin Microbiol.	1995	17

Figure 6 shows that since 1993, the research articles concern on # mycobacterium tuberculosis strain published by Alland D (1994) and Vanembden JDA (1993) in the journals NEW ENGL J MED and J CLIN MICROBIOL, respectively, have been widely cited, with a total of 36 and 28 citations. Subsequently, # methodological problem appeared in a large number of articles that were cited, among which Small PM, (1994), Kamerbeek J (1997), Van SOOLINGEND, (1999) published articles in NEW ENGL J MED, J CLIN MICROBIOL and J INFECT DIS, respectively, which became high Cited Articles. # Genetic diversity also appeared in a large number of cited articles from 2004 to 2016, which were published in J CLIN MICROBIOL and BMC MICROBIOL by Brudey K (2006) and Supply P (2006), respectively. Since 2013, # systematic review and #genexpert remnant-related articles are an important research basis for the molecular epidemiology of tuberculosis. The research related to # northern india since 2019 also provides important research information and helps in this field.

## Discussion

Based on the multidimensional quantitative analysis of the TB molecular epidemiology literature published from the establishment of the Web of Science database to the present, it can be noticed that the number of TB molecular epidemiology studies had gradually increased before 2010s. Although the number of related articles has shrunk in recent years, it is prospected that articles associated with treatment and prevention of tuberculosis may spring up with the development of molecular epidemiology and innovation of methodological problem.

In terms of the number of papers of institutions and countries and the frequency of citations, authors and institutions from developed countries in Europe and the United States have carried out more researches on the molecular epidemiology of tuberculosis, and their influence is also relatively huge. It is connected with not only economic factors but also epidemiological characteristics of tuberculosis. For the developed countries, there are definitely sufficient funds, qualified talent, and advanced equipment to support the research. Even more, the prevalence of tuberculosis is low and not many new positive cases of TB are discovered every year in various developed countries. Then they have the capacity to explore all of them. Furthermore, mainly family transmission is convenient for carrying out related research (8–10). However, for the developing countries, it is just the opposite with a high burden of tuberculosis but lack funds, technology and equipment. As a result, control of tuberculosis might deteriorate and it becomes a vicious cycle because of the imbalance. Our research shows that there is close cooperation among countries both in developed and developing countries. Developed countries provide funding and technical support in low-income developing countries with a high TB burden. In this regard, developing countries should strive for international cooperation and financial support including some non-governmental organization funds, such as Global Fund Project and Gates Foundation Tuberculosis Prevention and Control projects, which will help the development of scientific research and reduce this global health threat.

We have acknowledged some authoritative writers in the field of tuberculosis molecular epidemiology, such as RASTOGI N, D Alland, D, Small PM, etc, through the distribution of literature authors and citation analysis. The top two cited articles are published in the New England Journal of Medicine in 1994 by D Alland and Small PM. These two articles used DNA fingerprinting combined with traditional epidemiological methods to investigate the pathogenesis of recurrent pulmonary tuberculosis in the New York City community and the transmission mechanism and risk factors of tuberculosis in the city of San Francisco. It has been recognized by scholars to solve the problem that traditional tuberculosis epidemiology cannot determine whether tuberculosis patients are recently infected or reactivated latent infection and (11, 12). The important authors play a decisive role in promoting the development of the discipline and opening up the depth and breadth of the research field. Follow-up research in the field of TB molecular epidemiology can also be achieved by paying attention to the above-mentioned core authors.

A review of the relevant published literatures shows that molecular epidemiology is used to study tuberculosis susceptibility, the occurrence and spread of the disease, the relationship between infection and the incubation period of onset, the ratio of endogenous recurrence and exogenous relapse, whether different strains have different pathological manifestations, transmission patterns and differences in susceptibility of anti-tuberculosis drugs, frequency of mixed infections, risk factors for morbidity in different populations, monitoring of laboratory testing errors, etc. It plays an important role in the fields of preventive medicine and basic medicine, and even big data analysis of public health events. There are currently five molecular epidemiological methods used in MTB research: (1) Typing method based on a variable number of tandem repeats of MTB scattered repeat units (MIRU-VNTR); (2) spacer oligonucleotide typing method (Spoligotyping); (3) insertion sequence 6,110 (IS6110) restriction fragment length polymorphism (IS6110-RFLP); (4) single nucleotide polymorphism (SNP); (5) whole genome sequencing (WGS) technology. Among them, WGS has the advantages of high throughput, high accuracy, and convenience. It can obtain the whole genome sequence information of Mycobacterium tuberculosis, which can not only identify the species but also analyze the phylogenetic relationship between MTB, infection sources, and individual process of dissemination (13–19). Current molecular epidemiological surveillance methods also have their own shortcomings. For example, the detection process is slow due to dependence on MTB culture. Some method needs expensive equipment, large data analysis and standardization in technical expertise. Therefore, it is foreseeable that the continuous improvement and innovation of methods on molecular epidemiology of tuberculosis will be the focus and difficulty of research.

This study has certain limitations. First, the literature included in this study was all in English, and there was a language bias. Moreover, some recently published high-quality literature may be cited infrequently due to the short time of publication, which may lead to certain discrepancies between the research results and the real situation. Citation analysis is based on the Web of Science Core Collection, which may miss some important documents indexed by other databases, leading to biased results. Bibliometrics and visual analysis in medical research requires more comprehensive and up-to-date data and to overcome the adverse effects of the language and time of publication of the literature.

## Conclusion

At present, American and European countries are still in a dominant position in the field of molecular epidemiology of tuberculosis. The relevant literatures they published are among the top in the world in terms of quantity and quality. In recent years, the research has focused on the genetic polymorphisms and family characteristics of Mycobacterium tuberculosis for a long time. With the progress of molecular epidemiology and innovation of methodological problem, more articles associated with treatment and prevention of tuberculosis may be published in the future. More frequent and deeper cooperation among countries or institutions is essential. This paper reflects the development of the molecular epidemiology of tuberculosis through the perspective of bibliometrics. It has specific guidance and reference significance for institutions and scholars who carry out tuberculosis-related research.

## Data availability statement

The raw data supporting the conclusions of this article will be made available by the authors, without undue reservation.

## Author contributions

Z-yG and DC conceived and designed the analysis. M-qZ and X-xL wrote the paper. SL colledcted the data. Z-yR contributed data and analysis tools. RX performed the analysis and provided other contribution. All authors contributed to the article and approved the submitted version.

## Conflict of interest

The authors declare that the research was conducted in the absence of any commercial or financial relationships that could be construed as a potential conflict of interest.

## Publisher's note

All claims expressed in this article are solely those of the authors and do not necessarily represent those of their affiliated organizations, or those of the publisher, the editors and the reviewers. Any product that may be evaluated in this article, or claim that may be made by its manufacturer, is not guaranteed or endorsed by the publisher.
